# How Does Public Opinion Evolve in the Post-Truth Era? A Modeling and Simulation Study of Group Negative Emotion in Deviation Communities

**DOI:** 10.3390/bs16071097

**Published:** 2026-07-02

**Authors:** Jing Cao, Meng Yao, Haixiang Guo, Yudi Chen, Yulong Bao

**Affiliations:** 1 School of Public Administration, Xiangtan University, Xiangtan 411105, China; caojing1031@xtu.edu.cn (J.C.); 202305191132@smail.xtu.edu.cn (M.Y.); 202421100487@smail.xtu.edu.cn (Y.B.); 2 Research Center for Emergency Management, Xiangtan University, Xiangtan 411105, China; 3 School of Economics and Management, China University of Geosciences (Wuhan), Wuhan 430074, China; 4 Laboratory of Natural Disaster Risk Prevention and Emergency Management, China University of Geosciences, Wuhan 430074, China; 5 Hubei Key Laboratory of Environment and Culture in Yangtze Regions, China University of Geosciences, Wuhan 430074, China; 6 College of Economics and Management, Nanjing University of Aeronatics and Astranautics, Nanjing 211106, China; ychen55@nuaa.edu.cn

**Keywords:** public opinion deviation, group negative emotion, opinion dynamics, post-truth era, emergency

## Abstract

In the post-truth era, effective governance of emergency public sentiment faces significant challenges due to the phenomenon of opinion deviation. Although online public opinion has been extensively investigated, the specific impact of Public Opinion Deviation (POD) on the evolution of group negative emotion remains inadequately understood. To address this gap, this study proposes an explicable framework that integrates community detection, text mining, and opinion dynamics. Opinion deviation communities are identified by applying the Louvain algorithm and TextRank to social media data, followed by a deviation analysis of community topics against core issues. Subsequently, a multi-stage quantification model is constructed to measure the severity of POD. During these processes, we develop a novel Opinion Dynamics F-J model (POD F-J model) and its intervention-oriented model (IPOD F-J model), which incorporate the quantified POD severity to simulate the evolution of group negative emotion. Our findings demonstrate an intrinsic correlation between the severity of opinion deviation and the intensity of group negative emotion at the information level, thereby confirming the necessity of targeted intervention. Simulation experiments indicate that different intervention strategies should be adopted depending on the situation. Moreover, the application of a greedy algorithm identifies the time points corresponding to the peak severity of deviation and its onset as the efficiency-oriented intervention timings. This study provides a data-driven framework for monitoring and mitigating emotional contagion in deviation communities, contributing to both the theory and practice of digital governance.

## 1. Introduction

The term “post-truth” was named the 2016 Word of the Year by Oxford Dictionaries. In the post-truth era, appeals to emotion and personal belief shape public opinion more powerfully than objective facts (McIntyre, 2018). During public safety incidents, multiple factors increase the complexity and uncertainty of opinion dissemination, including the obstructive effect of emotional language (Yu et al., 2019), the hostile media effect (X. Zhang et al., 2023), and the public’s tolerance of misinformation (Effron & Helgason, 2022). In this context, the post-truth tendencies of the public become especially pronounced: the line between truth and emotion blurs, and emotional impulses override rational deliberation (Xiang, 2022). Such a situation may intensify emotional polarization, increase fragmentation in public discourse, and ultimately undermine social stability (X. Liu & Zhang, 2024). A shift in public attention from core issues to peripheral topics signals the presence of opinion deviation. Specifically, in emergency settings, public opinion deviation (POD) refers to the diversion of public attention away from the core issues that decision-making experts expect (i.e., topics that, through group discussion, could generate high-quality information for decisions) toward content of low relevance or minor significance. This phenomenon not only complicates the governance of public sentiment in the post-truth era but also injects informational noise into emergency decision-making, creating unnecessary risks (Cao et al., 2022). Therefore, understanding the evolution of negative emotions within POD groups and identifying effective intervention strategies is of great importance.

In public opinion research, some scholars have explored the applications of communication dynamics (J. Wang et al., 2024; G. Yang et al., 2024; X. Liu et al., 2025; X. Yang et al., 2025), game theory (Y. Xu et al., 2025; G. Xu et al., 2024), and greedy algorithms (Q. Zhang et al., 2025; Ding & Li, 2020) to public opinion management. Others have focused on users’ negative emotions on social media platforms (A. Chen et al., 2025; Yan et al., 2025). Still others have conducted sentiment-topic analyses of social media data related to extreme weather (R. Zhang & Chen, 2025), floods (C. Li et al., 2025), earthquakes (C. Wang et al., 2024), public health events (L. Wang et al., 2024; Lu et al., 2022), wars (Islam et al., 2025), and air crashes (Dai et al., 2024) to examine public opinion. Furthermore, several studies have produced valuable findings by using data to assist accident analysis (Khan et al., 2025; Zhou et al., 2024; Chang et al., 2022) and to facilitate public participation in emergency decision-making (Cao et al., 2022; Cheng et al., 2026; X. Chen et al., 2022; Zhu et al., 2023; X. Xu et al., 2023, 2024a, 2024b).

Given the diverse and highly complex nature of online public opinion during emergencies, regulators face growing pressure to develop simulation tools for opinion dynamics. As a wildly used approach for simulating the evolution of group opinions or emotions, opinion dynamics has drawn considerable scholarly attention. Yao et al. (2022) constructed a two-layer complex network model based on opinion dynamics to study topic resonance in online social networks. Xie et al. (2023) developed an opinion dynamics model tailored to the dual-hazard scenario of natural disasters and public health events. Inspired by the spiral of silence theory, H. Xu et al. (2023) proposed a new social Hegselmann–Krause (H-K) dynamics model based on the opinion climate. Drawing on an analysis of how different emotions emerge and interact in public risk events, M. Liu et al. (2025) built a negative emotion evolution model grounded in social comparison theory. Building on cognitive dissonance theory, Wei et al. (2023) developed an opinion evolution model from a psychological perspective. Recognizing that individuals’ opinions in online environments are jointly shaped by interpersonal networks and media information, they further improved the DW model (Wei et al., 2024). However, few scholars have examined the scenario of public opinion deviation from an opinion dynamics perspective, leaving a critical gap in public opinion governance.

The characteristics of group irrationality, especially as manifested in public opinion deviation, have become even more pronounced during emergencies in the post-truth era. Li highlighted the urgent need to guide and correct “negative tendencies” in public opinion expression in this era (B. Li, 2021). Liu et al. similarly stressed the importance of promptly reversing negative emotional trends and curbing emotional polarization (X. Liu & Zhang, 2024). The impact of the public opinion environment has begun to attract serious academic attention. For instance, Y. Wang et al. (2024) found that environmental stimulation can influence individual states. Other scholars have examined, separately, how public opinion perception affects behavioral decision-making and information dissemination (W. Li et al., 2023; H. Zhang et al., 2023). With regard to opinion deviation, H. Liu (2018) argues that it encompasses false media reports, public cognitive biases, and delayed responses to public sentiment. C. Yang (2020, 2021) conducted a quantitative study of public opinion deviation by calculating objective weights and explored the influence of emotion diffusion and information cascades on deviation. Ji and Wei (2021) simulated the process of public opinion deviation and found that highly authoritative “super-media” can induce such deviation. Su et al. (2025) also investigated whether entertainment events divert public attention from public affairs.

In summary, previous research has achieved notable results in the analysis of public opinion data and opinion dynamics. Some studies have further explored the causes, influencing factors, and governance of extreme negative emotions in the post-truth era. Nevertheless, to date, no study has accounted for the influence of public opinion deviation on group negative emotions in the post-truth era, leaving several key issues unresolved: (1) How can we accurately identify communities characterized by public opinion deviation? (2) How can we design a modeling framework for the evolution of negative emotions within such deviation communities? (3) How can we develop targeted intervention tools for group negative emotions in opinion deviation scenarios?

Against this backdrop, the present paper uses real social media data to identify POD communities, quantify the severity of POD at multiple stages, and incorporate that quantification as a parameter to improve the F-J model. The resulting POD F-J model simulates the evolution of negative emotions in POD communities. Based on this model, we construct an intervention-oriented model—the IPOD F-J model—by integrating a greedy algorithm. We then analyze differences in intervention effects and preferences for intervention timing across various strategies, thereby providing a data-driven tool for governments to effectively regulate public sentiment related to opinion deviation.

The remainder of this paper is organized as follows. Section 2 presents preliminaries that facilitate understanding of the proposed models. Section 3 describes the POD community identification model, the multi-stage quantification model for POD severity, the POD F-J model, and the IPOD F-J model. Section 4 applies these models to a real-world public safety incident. Section 5 concludes the paper.

## 2. Preliminary Work

### 2.1. Opinion Dynamics

Opinion dynamics offers deeper insights into how group opinions or emotional states evolve over time. Let ${\;x}_{t}^{i}\in \left[0,1\right]$ denote the negative emotion value of individual *i* (*i* ∈ {1, 2, 3, …, *n*}), where n is the total number of individuals) at stage *t* (*t* ∈ {1, 2, 3, …, *t_max_*}), where $t_{max}$ is the total number of stages). Thus, the matrix $X_{t}\;$=$\;{\left(x_{t}^{1},x_{t}^{2},\ldots ,x_{t}^{n}\right)}^{\Gamma }$ represents the negative emotion values of all individuals at stage *t*.

The DeGroot model (Degroot, 1974) is a classic model of opinion dynamics. In this model, an individual’s negative emotion is updated based on the negative emotions of their neighbors, as shown in Equation (1). Here, *W* is the influence weight matrix and $w_{ij}\in \left[0,1\right]$ denotes the influence weight of individual *i* on individual *j*.

(1) $$X_{t+1}=WX_{t}$$

The Friedkin–Johnsen (F-J) model (Friedkin & Johnsen, 1990), as shown in Equation (2), extends the DeGroot model by incorporating both individuals’ initial negative emotions and their openness to neighbors’ negative emotions, making it more realistic:

(2) $$X_{t+1}=AWX_{t}+(I\;-\;A)X_{1}$$

where the openness matrix *A* is an n × n diagonal matrix, and $a_{ii}\in \left[0,\;1\right]$ represents the degree to which individual *i* is influenced by the negative emotions of their neighbors. *I* is the identity matrix. $X_{1}$=${\left(x_{1}^{1},x_{1}^{2},\ldots ,x_{1}^{n}\right)}^{\Gamma }$ is the initial negative emotion matrix, representing the negative emotion values of all individuals at the initial stage *t* = 1.

It should be noted that this paper employs the opinion dynamics model to describe the evolution of group negative emotions. In the standard opinion dynamics model, an opinion value closer to 0 indicates a more negative stance, while a value closer to 1 indicates a more positive stance. Here, we reinterpret the opinion state as the negative emotion state. Thus, an opinion value closer to 0 corresponds to less negative emotion, whereas a value closer to 1 indicates more severe negative emotion.

### 2.2. Mamdani Fuzzy Inference System

**Figure 1:**
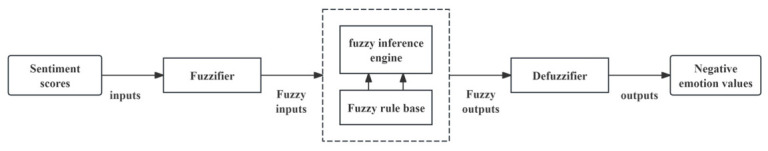
Mamdani fuzzy inference flowchart.

The Mamdani fuzzy inference system is widely used and consists of four components: a fuzzifier, a fuzzy inference engine, a fuzzy rule base, and a defuzzifier. The system first fuzzifies the input text sentiment scores using appropriate membership functions, then performs inference based on the predefined fuzzy rules, and finally applies a suitable defuzzification method to convert the fuzzy output back to a precise value. This process transforms precise numerical inputs into fuzzy values, applies fuzzy reasoning, and then returns a crisp output—thereby yielding the negative emotion value of the text. The overall procedure is illustrated in Figure 1.

### 2.3. Text Feature Extraction Method

#### 2.3.1. Text Rank

TextRank is a graph-based keyword extraction method derived from PageRank. It splits a document into sentences, performs word segmentation and filtering to obtain a candidate set of keywords, and then constructs a graph model. In this graph, nodes represent candidate keywords, and edges represent co-occurrence relationships between them. The extracted keywords can serve as a basis for community classification. By treating words as nodes and linking them through co-occurrence, TextRank effectively captures semantic information between words, thereby providing a deeper understanding of the text content. In contrast, keyword extraction algorithms such as TF-IDF perform poorly in semantic understanding (F. Zhang & Song, 2024).

#### 2.3.2. Louvain Algorithm

The Louvain algorithm is a community detection method that uses a heuristic, greedy optimization strategy to maximize modularity in complex networks. It identifies topic-based discussion communities within emergency-related online public opinion by iteratively optimizing the modularity of a keyword co-occurrence network. Modularity is a metric that measures the quality of community partitioning, reflecting both the density of connections within communities and the sparsity of connections between them. The algorithm first increases modularity by moving individual nodes locally, then merges adjacent communities to form a new network, and repeats this optimization process until no further improvement in modularity is possible. Compared with the GN algorithm, the LPA algorithm, and FCM clustering analysis, the Louvain algorithm achieves higher accuracy and effectiveness in community division (Luo et al., 2024).

#### 2.3.3. Word2vec

Word2vec is an NLP tool introduced by Google in 2013. It is an unsupervised model that learns semantic knowledge from large text corpora. By vectorizing words, it facilitates the quantitative measurement of relationships between community keywords and core issue keywords, and allows for the exploration of their potential associations.

#### 2.3.4. Snow NLP

SnowNLP sentiment analysis is a method for recognizing sentiment in Chinese text based on the SnowNLP programming library. Its goal is to convert text from social media platforms such as Sina Weibo into quantifiable values, thereby facilitating subsequent sentiment understanding and analysis. This method first performs Chinese word segmentation on the original text, then identifies and analyzes words with emotional tendencies to determine their positive or negative connotations. Using a sentiment dictionary and a statistical model, the system ultimately computes a sentiment score for each text. The score typically ranges between 0 and 1: the closer the score is to 1, the more positive the sentiment; conversely, the closer it is to 0, the more negative the sentiment.

#### 2.3.5. BERT

BERT is a deep learning-based pre-trained language model that uses the Transformer architecture to capture contextual information and semantic relationships in text. By undergoing unsupervised pre-training on large-scale text corpora, it acquires rich linguistic features. Fine-tuned BERT can then be adapted to specific text classification tasks.

### 2.4. Greedy Algorithm

The greedy algorithm is a heuristic method for solving optimization problems. At each step, it selects the candidate solution that maximizes the objective function. The core idea is to gradually build a globally efficiency-oriented solution through a series of locally optimal choices. In the context of selecting intervention timings to reduce the average negative emotion value of a group, the algorithm picks the time point that yields the largest reduction. The key characteristic of the greedy algorithm is that it makes the efficiency-oriented decision at each step without considering the potential impact on future steps.

## 3. Method Principle

### 3.1. Overall Methodological Framework

This study is grounded in opinion dynamics. By constructing a group negative emotion evolution model that integrates public opinion deviation (POD) severity, sensitivity, and intervention effects, we investigate the evolution of negative emotions within POD communities and the role of intervention strategies.

First, a POD community identification model is established through data acquisition, preprocessing, topic community classification, core issue definition, and community deviation analysis. A comparative analysis is also conducted between the evolution of negative emotions in POD communities and that in normal communities.

Second, a multi-stage quantification model for POD severity is developed by combining the proportion of posts and comments from POD communities at each stage with the normalized total number of posts and comments during the same period.

Third, a negative emotion evolution model for POD communities is constructed by incorporating the intensity of negative emotional stimuli, which is derived from both POD severity and sensitivity. Building on this, an intervention scalar that includes parameters such as intervention strength and intervention persistence is introduced for simulation experiments.

**Figure 2:**
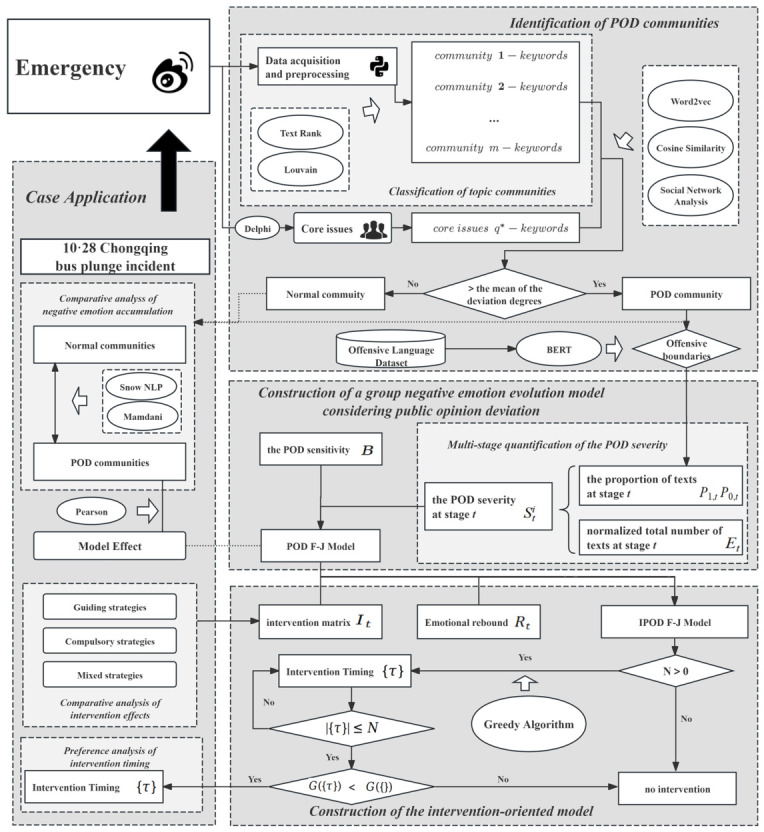
The flowchart of the proposed method.

Finally, the greedy algorithm is used to identify the optimal intervention strategy and intervention timing preferences. The flowchart of the proposed method is shown in Figure 2.

### 3.2. POD Community Identification

#### 3.2.1. Data Acquisition and Preprocessing

During the evolution of online public opinion in emergencies, there are notable differences in both the content of discussions and the intensity of negative emotions expressed. First, data mining techniques are applied to collect textual data, which then undergo cleaning. To directly extract the intensity of negative emotions from the text, we combine SnowNLP sentiment analysis with the Mamdani fuzzy inference method, aiming to make the output more focused on negative emotional intensity. The sentiment scores generated by SnowNLP are used as input. After fuzzification using triangular membership functions, fuzzy inference based on predefined rules, and defuzzification via the centroid method, the negative emotion value is obtained. The predefined fuzzy rule base is presented in Table 1.

**Figure 3:**
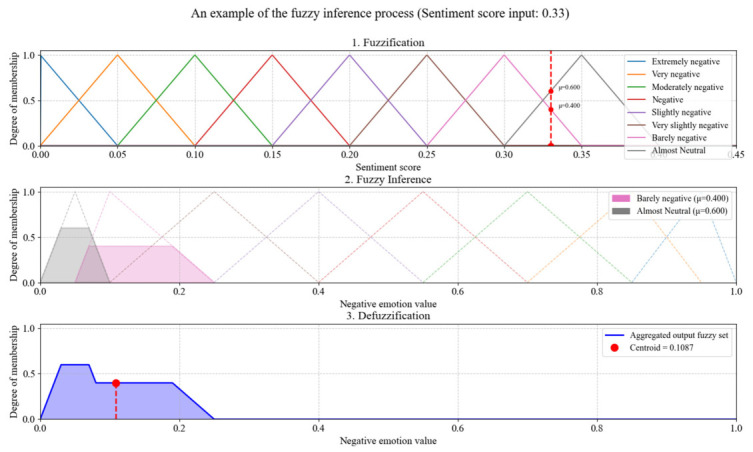
An example of the fuzzy inference process.

Under these rules, when the text sentiment score falls within the negative emotion interval, a lower sentiment score results in a higher negative emotion output from the fuzzy inference system. In this study, the sentiment score range corresponding to negative emotion is set to [0, 0.4]. For sentiment scores outside this interval, the negative emotion output is set to zero. Figure 3 provides an example of the fuzzy inference process. When the input sentiment score is 0.33, the resulting negative emotion value is 0.1087.

#### 3.2.2. Topic Community Classification

In topic community classification, we integrate the TextRank and Louvain algorithms. First, keywords are extracted from each text using TextRank, and meaningless words are removed. Next, keywords that appear 200 times or more are selected to construct a co-occurrence network. Social network analysis is then used to compute the degree centrality of each keyword. After that, the selected high-frequency keywords are processed with the Louvain community detection algorithm, which divides them into communities through modularity optimization, yielding the keyword set for each community. The preprocessed full dataset is used for community classification to identify topic communities across the entire timeline and to assign community membership labels to each text. There are m topic communities in total. It is important to note that posts or comments that contain no keywords or lack obvious topic-assigning keywords do not convey explicit topic-related information. Such texts fall outside the scope of this study and are not included in the topic communities.

#### 3.2.3. Deviation Analysis and POD Community Determination

After obtaining the community information of each text, begin analyzing the deviation degree of community topics. Communities whose discussion content substantially deviates from the core issues are identified as public opinion deviation (POD) communities.

**Definition** **1.** *Let* $O^{q}$ *be the mean vector of the representative keywords in community q* $(q\in \left\{1,2,3,\ldots ,m\right\})$ *processed by the Word2vec algorithm. We identified the top four keywords by degree centrality within each community as its representative keywords.* $q^{*}$ *is the set of core issue keywords established by experts based on the “Provisions on Ecological Governance of Network Information Content” (Cyberspace Administration of China, 2019). The formulation basis of the “Provisions on Ecological Governance of Network Information Content” includes the “National Security Law of the People’s Republic of China,” the “Cybersecurity Law of the People’s Republic of China,” and other higher-level laws, and it has compulsory force at the implementation level. The “Provisions on Ecological Governance of Network Information Content” is one of the most fundamental and core administrative regulations in current Chinese Internet governance. It serves as the decision-making basis for expert selection of core topic keywords in this article*.

The core issue keywords were identified through a group decision-making (GDM) process using a Delphi method. Group experts were invited to participate, each holding a doctoral degree in emergency management, public opinion governance, or law, or possessing at least five years of practical experience in emergency response or public opinion regulation. The experts were first presented with the contextual information of the Chongqing bus plunge incident and the relevant decision-making principles (Cyberspace Administration of China, 2019). They were then asked to independently propose keywords representing the core issues that, in their view, should guide public discussion and decision-making during the emergency. After collecting the initial responses, the research team aggregated the keywords and circulated the aggregated list to the experts for a second round of review and refinement. The Delphi method helps mitigate individual expert bias by combining multi-round anonymous feedback, thereby increasing the validity of the core issue definition. Meanwhile, providing decision-making guidelines and specifying the decision scenario can help mitigate experts’ biases when selecting core issue keywords.

Now, let $F^{q}$ be the cosine similarity between the community vector $O^{q}$ and the core issue vector $O^{*}$ (the mean vector of all core issue keywords). The deviation degree ${Deviation}^{q}$ is then derived as shown in Equations (3) and (4), where $O_{l}^{q}$ and $O_{l}^{*}$ are the values of the vectors $O^{q}$ and $O^{*}$ in the l-th dimension $\left(l\;\in \;\left\{1,\;2,\;3,\;\ldots ,\;l_{max}\right\}\right)$. For computational efficiency, a dimensionality of 100 is commonly used because it captures most of the useful signal (Mikolov et al., 2013); therefore, $l_{max}$ is set to 100.

(3) $$F^{q}=\frac{O^{q}\cdot O^{*}}{\left\|O^{q}\right\|\left\|O^{*}\right\|}=\frac{\sum_{l=1}^{l_{max}}O_{l}^{q}O_{l}^{*}}{\sqrt{\sum_{l=1}^{l_{max}}{\left(O_{l}^{q}\right)}^{2}}\cdot \sqrt{\sum_{l=1}^{l_{max}}{\left(O_{l}^{*}\right)}^{2}}}$$

(4) $${Deviation}^{q}=1\;-\;F^{q}$$

A community is identified as a POD community only if its topic deviation degree exceeds a threshold. In this paper, the threshold is set as the average topic deviation degree across all communities. A community whose deviation degree exceeds this threshold is considered to have discussion content that deviates from the core issues more than typical. If community *q* meets this criterion and belongs to the overall community set *Q*, it is then classified into the POD community set *Q_POD_* (*Q_POD_* ⊆ *Q*), as shown in Equation (5). Otherwise, community *q* is regarded as a normal community within Q.

(5) $${Deviation}^{q}>\frac{\sum_{q=1}^{m}{Deviation}^{q}}{m}\Rightarrow \;q\in \;Q_{POD}$$

The above formula only captures semantic deviation and does not account for differences in the nature of the discussion within deviated communities; for instance, some may exhibit offensive boundaries. Therefore, from the perspective of offensive boundaries, we further classify opinion deviation communities into two types: those with offensive boundaries, denoted as $Q_{POD}^{1}$, and those without, denoted as $Q_{POD}^{0}$.

Offensiveness is a sociological mechanism of “othering” and structural scapegoating. Using a fine-tuned BERT model, we identify offensive texts in each POD community. For each community *q*, we then construct two keyword co-occurrence networks using only its offensive texts and only its non-offensive texts, respectively. The density of a network determines how structured the discussions are, and the weighted clustering coefficient is a commonly used measure of this property. Following Barrat et al. (2004), we compute the average weighted clustering coefficient ${\bar{C}}^{w}$ for each network. For a node *i* with degree $k_{i}\;\geq \;2$, its weighted clustering coefficient is $c_{i}^{w}=\frac{1}{s_{i}\left(k_{i}-1\right)}\sum_{j,h}\frac{\omega_{ij}+\omega_{ih}}{2}\cdot {adj}_{ij}\cdot {adj}_{ih}\cdot {adj}_{ih}$, where $\omega_{ij}$ is the co-occurrence count of keywords *i* and *j* (edge weight), $s_{i}=\sum_{j}{adj}_{ij}\cdot \omega_{ij}$ is the node strength, ${adj}_{ij}=1$ if an edge exists between *i* and *j* and 0 otherwise, and the summation runs over all unordered pairs of neighbors (*j*, *h*) that are themselves connected. The network’s ${\bar{C}}^{w}$ is the average of $c_{i}^{w}$ over all nodes with $k_{i}\;\geq \;2$. Let ${\bar{C}}_{off,q}^{w}$ and ${\bar{C}}_{non,q}^{w}$ denote the values for the offensive and non-offensive networks, respectively. A community is considered to have a structurally cohesive offensive pattern if ${\bar{C}}_{off,q}^{w}>{\bar{C}}_{non,q}^{w}$.

In addition, according to the Pareto principle, prioritizing the control of the more severely problematic groups can effectively manage the overall public opinion. This study focuses on the more problematic groups, i.e., those with a higher proportion of offensive texts. The proportion of offensive posts in a community can serve as a partial indicator of its level of offensiveness, while the average proportion of offensive texts across all deviated communities is used as the classification threshold. We calculate the proportion of offensive texts in each POD community, denoted as ${Offense}^{q}$. A community is classified as having offensive boundaries ($Q_{POD}^{1}$) if it satisfies both of the following conditions: (i) ${\bar{C}}_{off,q}^{w}>{\bar{C}}_{non,q}^{w}$, and (ii) ${Offense}^{q}$ exceeds the average proportion across all POD communities, as defined in Equation (6), where $m_{POD}$ is the number of opinion deviation communities. Otherwise, it belongs to $Q_{POD}^{0}$.

(6) $${\bar{C}}_{off,q}^{w}>{\bar{C}}_{non,q}^{w}\;\mathrm{and}\;{Offense}^{q}>\frac{\sum_{q\in \;Q_{POD}}{Offense}^{q}}{m_{POD}}\Rightarrow \;q\in \;Q_{POD}^{1}$$

The weighted clustering coefficient captures the structural cohesiveness of a keyword co-occurrence network, revealing whether offensive posts form densely interconnected topical clusters. We first apply this network-based absolute criterion to each POD community: if ${\bar{C}}_{off,q}^{w}>{\bar{C}}_{non,q}^{w}$, the community is considered to have a structurally cohesive offensive pattern. Only those communities that satisfy this condition then proceed to the relative proportion threshold test.

The proportion of offensive texts measures the relative severity of offensive discourse within a community. Relying solely on the relative proportion threshold (Equation (6)) may, however, become ineffective under special circumstances—for example, when the overall public opinion environment is highly toxic and most communities exhibit similarly high proportions of offensive content. In such cases, expert judgment can be invoked as a complementary recourse.

### 3.3. Evolution Model of Group Negative Emotion Considering POD

#### 3.3.1. Multi-Stage Quantification of POD Severity

At different stages of public opinion evolution, the severity of POD varies due to factors such as shifts in public attention and fluctuations in public opinion intensity. To quantify POD severity at each stage, the proposed model selects two key parameters: (1) the proportion of posts and comments (in this paper, posts and comments are both treated as texts) from POD communities at each stage, and (2) the normalized total number of posts and comments during the same period. The first parameter reflects how much public attention shifts toward POD topics at each stage, while the second reflects the overall intensity of public opinion during that stage. By analyzing these two dimensions, the model provides a more comprehensive measure of POD severity. Based on this, the paper proposes the following model.

**Definition** **2.** *Let* $P_{1,t}$ *and* $P_{0,t}$ *denote the proportions of texts from POD communities with and without offensive boundaries, respectively, at stage t. Their calculation methods are given in Equations (7) and (8), where* $e_{t}^{q}$ *represents the number of posts and comments in community q at stage t*.

(7) $$P_{1,t}=\frac{\sum_{q\in {\;Q}_{POD}^{1}\;}e_{t}^{q}\;}{\sum_{q\in \;Q}e_{t}^{q}\;}$$

(8) $$P_{0,t}=\frac{\sum_{q\in \;Q_{POD}^{0}\;}e_{t}^{q}\;}{\sum_{q\in \;Q}e_{t}^{q}\;}$$

**Definition** **3.** $E_{t}$ *is the normalized total number of posts and comments at stage t, whose calculation method is shown in Equation (9)*.

(9) $$E_{t}=\frac{\sum_{q\in \;Q\;}e_{t}^{q}\;}{\sum_{t=1}^{t_{max}}\sum_{q\in \;Q}e_{t}^{q}\;}$$

**Definition** **4.** *Let* $S_{t}$ *be the POD severity matrix at stage t, which is an* n×1 *matrix. The element* ${\;S}_{t}^{i}$ *represents the POD severity experienced by individual i. Individuals in POD communities with offensive boundaries and those in POD communities without such boundaries are exposed to different levels of opinion deviation intensity, which is determined by* ${\;P}_{1,t},P_{0,t}$, *and* $E_{t}$*. The calculation method is shown in Equation (10)*.

(10) $$S_{t}^{i}=\left\{\begin{array}{cc} E_{t}\times P_{1,t} & if\;individual\;i\;belongs\;to\;Q_{POD}^{1}\\ E_{t}\times P_{0,t} & if\;individual\;i\;belongs\;to\;Q_{POD}^{0} \end{array}\right.$$

#### 3.3.2. The POD F-J Model

Based on the classical F-J model (Equation (2)), this paper introduces the POD sensitivity matrix *B* and the POD severity matrix $S_{t}$ and constructs a negative emotion evolution model for POD communities (POD F-J model), as shown in Equation (11), where $X_{1}\;=\;BS_{1}$ (Xie et al., 2023).

(11) $$X_{t+1}=\left(I-B\right)\left[AWX_{t}+\left(I-A\right)X_{1}\right]+BS_{t+1}$$

Here, the POD sensitivity matrix *B* is an n × n diagonal matrix with entries $b_{ii}\in \left[0,1\right]$. From a psychological perspective, individuals differ in the extent to which they are influenced by negative emotions arising from POD. According to cognitive appraisal theory, emotions are not directly determined by a stimulus situation; rather, they result from an individual’s subjective evaluation and cognitive processing of the stimulus. Because cognitive appraisals vary across individuals, the same POD event can trigger different levels of negative emotional response in different people. This is why matrix *B* is introduced.

The POD severity values are derived from real-world case data using the multi-stage quantification model described earlier. At a given stage, the severity value is assumed constant across individuals. The term $BS_{t}$ represents the amount of negative emotional stimulation caused by public opinion deviation at stage *t*. According to cognitive resource theory, an individual’s cognitive resources are limited; therefore, the complementary term $\left(I-B\right)$ is applied to the remaining part of the model (i.e., the part that evolves based on social influence and initial emotions). The meanings of all other matrices are the same as in the classical F-J model presented in Section 2.1.

### 3.4. Intervention-Oriented Model for POD Groups

#### 3.4.1. Analysis of Negative Emotion Intervention Strategies

In this study, the pressure stemming from POD serves as a stressor that influences group negative emotions. To maintain the stability of the public opinion ecosystem, we apply interventions to the individual negative emotion update process, specifically targeting the negative emotional stimulation caused by POD. These interventions may include measures such as reducing the severity of POD or decreasing group sensitivity to it. Based on existing research (Ren & Chao, 2021), public opinion intervention strategies can be classified according to the government’s “degree of compulsion” into three types, listed in ascending order: guiding, mixed, and compulsory. These strategies differ along two dimensions: immediate intervention strength and intervention persistence. Specifically, the guiding strategy has relatively low immediate strength but the highest persistence. Consequently, it is assigned a lower strength value and a higher persistence value. In contrast, the compulsory strategy exhibits the strongest immediate effect but the lowest persistence, leading to a higher strength assignment and lower persistence. The mixed strategy shows moderate performance on both dimensions, and its parameters are set accordingly to medium levels.

#### 3.4.2. The IPOD F-J Model

Following a thorough analysis of negative emotion intervention strategies, a model was constructed to illustrate the evolution of negative emotions within POD communities under the influence of these strategies.

**Definition** **5.** *Let* $I_{t}$ *denote the intervention effect on the evolution of negative emotions within the POD community at stage t.* $I_{t}$ *is a scalar, representing the intervention effect on the negative emotional stimulation caused by POD. In practice, excessively strong interventions may trigger psychological reactance. Therefore, we introduce* $R_{t}$ *to represent the additional negative emotion increment resulting from such reactance. The intervention-oriented model (IPOD F-J model) is given by Equation (12), where* $X_{1}\;=\;I_{1}BS_{1}+R_{1}$.

(12) $$X_{t+1}=(I-B)[AWX_{t}+\left(I-A\right)X_{1}]+I_{t+1}BS_{t+1}+R_{t+1}$$

**Figure 4:**
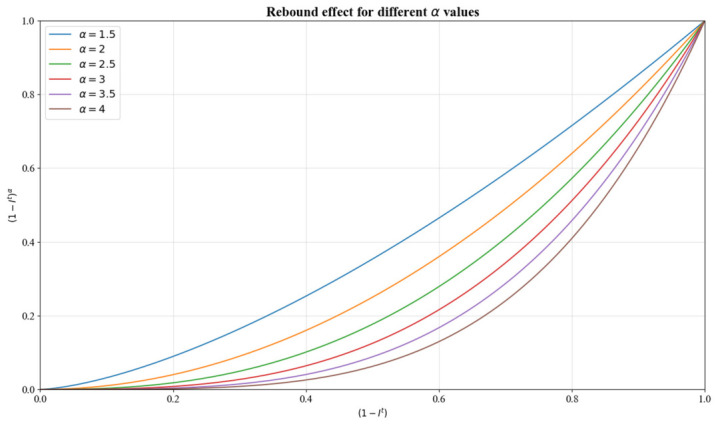
Rebound effect for different α values.

Here, $R_{t+1}={(1-I_{t+1})}^{\alpha }BS_{t+1}$, $\left(1-I_{t+1}\right)BS_{t+1}$ reflects the negative emotion value from the reduced POD stimulation after intervention. The exponent α (with α > 1) is an emotional rebound index that captures the heterogeneity of psychological reactance to intervention strength. A larger α indicates that the group is less sensitive to weak interventions (i.e., the rebound effect is more pronounced at high intervention strengths). Figure 4 illustrates this effect for different values of α (e.g., 1.5, 2.0, 2.5, 3.0, 3.5, 4.0).

The intervention effect $I_{t}$ depends on the intervention timing τ (where *τ* (*τ* ∈ {1, 2, 3, …, *t_max_*}) the immediate intervention strength, *Strength* $\in \left[0,1\right]$, and the intervention retention, *Retention* $\in \left[0,1\right]$. The specific rules are given in Equation (13). The values of *Strength* and *Retention* are determined by the type of intervention strategy.

The value of the intervention effect is affected by the intervention timing (*τ* ∈ {1, 2, 3, …, *t_max_*}), the immediate intervention strength, *Strength* $\in \left[0,1\right]$ and the intervention retention, *Retention* $\in \left[0,1\right]$. The detailed rules are presented in Equation (13). The values of *Strength* and *Retention* are related to the type of intervention strategy.

When *t* is an intervention moment, $I_{t}$ directly equals 1 − Strength.

When *t* is not an intervention moment but at least one intervention has already occurred, $I_{t}$ is set to 1 minus the maximum of the decayed intervention effects from all previous interventions.

When no intervention has occurred yet, or when *t* precedes the earliest intervention,${\;I}_{t}$ = 1 (i.e., no intervention effect).

(13) $$I_{t}=\left\{\begin{array}{cc} 1-Strength, & if\;\{\tau \}\neq \emptyset ,\;t\in \{\tau \}\\ 1\;-\underset{\tau \in \{\tau \},\tau \leq t}{\max}\left(Strength\;\times \;{Retention}^{t-\tau }\right), & if\;\{\tau \}\neq \emptyset ,\;t\notin \{\tau \},\;t\geq min(\tau )\\ 1, & if\;\{\tau \}\neq \emptyset ,\;t<min(\tau )\\ 1, & if\;\{\tau \}=\emptyset \end{array}\right.$$

#### 3.4.3. Identification of Efficiency-Oriented Intervention Timings

After identifying the POD communities, quantifying POD severity at stages, and constructing the IPOD F-J model, the efficiency-oriented intervention timing set $\left\{\tau \right\}$ and the number of interventions $\left|\left\{\tau \right\}\right|$ are selected based on the greedy algorithm under *N*, the upper limit on the number of effective interventions (*N* ∈ {0, 1, 2, …, *t_max_*}). The objective is to minimize G, the average negative emotion value over the entire time horizon.

Combining Equations (12)–(14), we build a model that identifies the efficiency-oriented intervention timing set $\left\{\tau \right\}$. Assuming that the values of *Strength* and *Retention* are predetermined, the selection of intervention time points is transformed into an optimization problem that minimizes the objective function, so that for each parameter combination the simulation output (the average negative emotion over all stages) reaches its optimum for comparison. The pseudocode for selecting intervention timings based on the greedy algorithm is shown in Algorithm 1. Considering that the objective function has not been proven to be submodular and therefore the greedy algorithm can only guarantee a locally optimal solution, we provide a comparative analysis between the greedy algorithm and exhaustive enumeration in Appendix A. The results show that the difference between the two methods is negligibly small and thus acceptable. Moreover, taking into account the computational efficiency and decision stability required in emergency scenarios, we adopt the greedy algorithm to determine the intervention timings.

(14) $$\left\{\begin{array}{c} Argmin\;G\left(\left\{\tau \right\}\right)=\frac{1}{t_{max}}\frac{1}{n}\sum_{t=1}^{t_{max}}\sum_{i=1}^{n}x_{t}^{i}\\ s.t.|\{\tau \}|\leq N \end{array}\right.$$

**Algorithm 1.** Greedy Algorithm for Efficiency-Oriented Intervention Timing Identification**Input:** $t_{\max},N,n,S_{t},B,A,W,Strength,Retention,\alpha$ **Output:** {τ}*, G* 1: $\{\tau {\}}^{*}\leftarrow \emptyset$ 2: Simulate with {τ}=∅ using IPOD F-J (Equation (12)) to obtain $G^{*}$ 3: **for** *k* = 1 to *N* **do** 4:      best_G ← ∞, best_time ← NULL 5:      **for** candidate = 1 to *t*_max_ **do** 6:           **if** candidate ∉ $\{\tau {\}}^{*}$ **then** 7:                $\{\tau {\}}_{\mathrm{test}}$ ← $\{\tau {\}}^{*}$ ∪ {candidate} 8:                Simulate using IPOD F-J (Equation (12)) with $\{\tau {\}}_{\mathrm{test}}$ to get $G_{\mathrm{test}}$ 9:                **if** G_test_ < $G^{*}$ **and** G_test_ < best_G **then** 10:                 best_G ← G_test_ 11:                 best_time ← candidate 12:              **end if** 13:         **end if** 14:     **end for** 15:     **if** best_time ≠ NULL **then** 16:          $\{\tau {\}}^{*}$ ← $\{\tau {\}}^{*}$ ∪ {best_time} 17:          $G^{*}$ ← best_G 18:    **else** 19:          **break** 20:    **end if** 21: **end for** 22: **return** $\{\tau {\}}^{*}$, $G^{*}$

## 4. Case Application

### 4.1. Case Selection and Data Acquisition

The Chongqing bus plunge incident that occurred on 28 October 2018, ranked among the top ten social-life buzzwords of that year. It is a highly representative public opinion deviation (POD) event with significant influence in the post-truth era. Ideally, public attention should have focused on objectively describing the accident and expressing concern for rescue efforts. However, in the early stage of the incident, public opinion shifted to blaming the female driver for “driving in the wrong direction” while wearing high heels. Due to multiple factors, public opinion frequently strayed from the core issues and triggered a severe public sentiment crisis. This study therefore selects the “Chongqing bus plunge incident on 28 October” as a real-world case. Using keywords such as “重庆公交坠江 (Chongqing bus plunge)”, “重庆女司机逆行 (Chongqing female driver driving in the wrong direction)”, “重庆女司机高跟鞋 (Chongqing female driver wearing high heels)”, “重庆公交女乘客 (Chongqing female bus passenger)”, “重庆司机错觉 (Chongqing driver’s illusion)”, “重庆司机疲劳驾驶 (Chongqing driver driving while fatigued)”, and “重庆学生幸存 (Chongqing student surviving)” (Kashgar Cyber Police, 2018), we collected data from the Sina Weibo platform covering the period from 0:00 on 28 October 2018, to 24:00 on 7 November 2018. After removing duplicate posts from the same user at the same time, 66,201 text entries were obtained and subsequently cleaned. The negative emotion value of each text was then derived using the Mamdani fuzzy inference system.

### 4.2. POD Community Identification in the Case

Topic community classification was performed on the preprocessed data. To partition the topic communities, we combined the TextRank and Louvain algorithms. Low-frequency keywords (word frequency < 200) as well as high-frequency but less informative terms such as “事件” (event) and “事情” (thing) were removed. A keyword co-occurrence network was then constructed based on the remaining keywords. Based on the results of social network analysis, keywords were ranked in descending order of degree centrality. The community division results are shown in Table 2, with a total of seven communities (*m* = 7). Within each keyword set, the higher the degree centrality of a keyword, the more important it is within the community, and the earlier it appears in the community’s keyword list. To label the topic communities, the first four keywords are used, as they represent the core content of each community.

When assigning a topic label to a specific text, texts that contain no extracted keywords or whose extracted keywords do not include any community keywords are marked as lacking a topic and are excluded from this study. Such texts are not considered in subsequent analyses, such as the calculation of negative emotion accumulation or the quantification of POD severity. As for the labeling process, the keywords of a text (including both community keywords and non-community keywords) are matched sequentially, and the community affiliation of the text is determined by a weighted voting method based on the degree centrality of the keywords. Subsequently, the top four keywords in each community were vectorized using Word2Vec to obtain the mean keyword vector for that community.

During the expert consultation, we first laid out the details of the Chongqing bus plunging into the Yangtze River: out of nowhere, a bus carrying multiple passengers broke through a guardrail and fell into the river while driving. The scene descended into traffic chaos, while social media quickly filled with rumors and emotional outbursts. Reliable information was scarce and hard to distinguish from misinformation. Both online and offline spaces were caught in extreme uncertainty and turmoil. Against this backdrop, the study focused on three key decision-making areas: incident assessment, emergency rescue, and public opinion guidance. Based on this, the experts discussed and finalized a set of core keywords: {重庆 (Chongqing), 公交车 (bus), 坠入 (fall into), 江中 (river), 悲剧 (tragedy), 祈祷 (pray), 希望 (hope), 逝者 (the deceased), 安息 (rest in peace), 关注 (attention), 救援 (rescue)}. This set captures the essence of the issue—objective facts about the accident, concern over rescue efforts, and mourning and prayers for the victims. We then calculated the cosine similarity between the community vector and the mean vector of this keyword set.

Specifically, the core keywords consist of: 重庆 (Chongqing), 公交车 (bus), 坠入 (fall into), and 江中 (river), which provide an objective account of the incident; 悲剧 (tragedy), 祈祷 (pray), 希望 (hope), and 逝者 (the deceased), which express concern for the victims; and 关注 (attention) and 救援 (rescue), which reflect concern for accident management. All these keywords align with the fifth point of Article 5 of the “Provisions on Ecological Governance of Network Information Content,” which states that “effectively responding to social concerns, dispelling doubts, analyzing events, and clarifying principles is conducive to guiding the public toward consensus,” as well as with the seventh point of the same article, which states that “content that emphasizes taste, style, and responsibility upholds truth, goodness, and beauty while fostering unity and stability.”

Based on this, the topic deviation degree of each community was quantified. The POD community identification model proposed in this paper (specifically Equations (3)–(5)) was then used to identify communities exhibiting public opinion deviation. The results show that Community 3 (“accident–female driver–news–driving in the wrong direction”), Community 4 (“driver–passenger–reason–situation”), Community 5 (“family members–salvage–victims–discovery”), and Community 7 (“China–location–Changsha–music”) are all POD communities. The detailed identification results are presented in Table 3.

**Figure 5:**
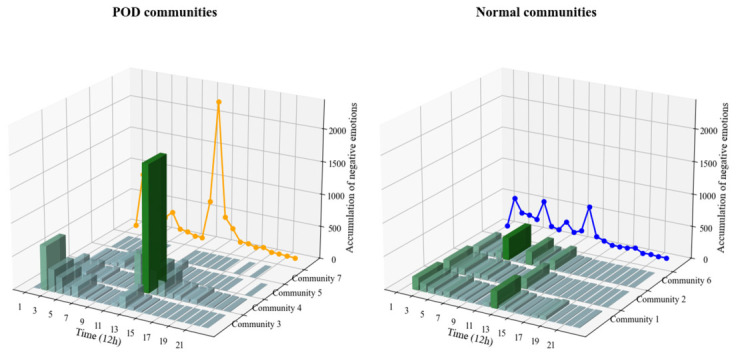
Comparison analysis between POD communities and normal communities.

On this basis, Figure 5 compares the accumulation of negative emotions between POD communities and normal communities over time. In this figure, the bars show the negative emotion accumulation for each community in each time period, while the line chart represents the total accumulation for each type of community over time. POD communities exhibit extreme negative emotion accumulation, characterized by alternating sudden surges. Specifically, negative emotions in Community 3 (“accident–female driver–news–driving in the wrong direction”) grow rapidly in the early stage of the emergency, whereas Community 4 (“driver–passenger–reason–situation”) shows a higher growth rate in the middle stage. In contrast, normal communities display relatively smooth fluctuations. These findings highlight the urgent need to develop a targeted decision support tool to intervene in the negative emotions of POD community groups.

Not all public opinion deviation (POD) communities share the same fundamental nature. Although both Community 3 (“accident–female driver–news–driving in the wrong direction”) and Community 7 (“China–location–Changsha–music”) deviate semantically from the core issues (i.e., objective description of the accident, rescue progress, and mourning for the victims), the social risks they pose are fundamentally different. For example, Communities 3 and 4 exhibit sudden surges in negative emotion, whereas Communities 5 and 7 do not. Moreover, we find that some communities contain strongly gender-offensive language. Examples from Community 3 include: “马路杀手女司机” (female driver, a road killer), “为什么女司机总是那么无脑” (why are female drivers always so brainless), and “以后法律要规定要增加一条严禁女司机开车” (the law should add a provision strictly prohibiting women from driving). Examples from Community 4 include: “我觉得司机正当防卫是正确的只能说泼妇就是泼妇” (I think the driver was justified in defending himself; let’s just say a shrew is a shrew), and “估计都是纷纷指责司机怎么可以打女人现在女的真特么跟黄帝一样” (people are all condemning the driver for hitting a woman, but nowadays women act as if they were the emperor).

The deviation in Community 7 mainly involves off-topic discussions of regional or entertainment matters. Its risk lies primarily in informational noise that dilutes public attention, without constituting systematic attacks on any specific group. In contrast, Community 3 constructs offensive boundaries: a large number of texts unilaterally blame “female drivers” for the accident, using offensive expressions such as “road killer,” “brainless,” and “the law should ban women from driving,” thereby forcibly associating women with negative labels such as “poor driving skills.” Community 4 reinforces stereotypes of women as “emotional” and “irrational” through derogatory terms like “shrew” and “women acting as if they were the emperor.” This gender-based scapegoating mechanism creates an offensive boundary against women.

Such offensive boundaries construct “women” as a “dangerous group,” reinforcing gender stereotypes and intensifying gender-based antagonism in online spaces. This kind of discourse not only harms the stigmatized group but may also erode social trust, degrade the quality of public discussion, and even trigger offline offensive behavior. In the long run, social cohesion is undermined.

From a data analysis perspective, we fine-tuned a BERT model on a gender-offensive language dataset (Deng et al., 2022) for gender-based offensive language detection. The training set consisted of 6579 annotated texts, with 1551 texts in the test set and 1657 in the validation set. The fine-tuned BERT model was then applied to the texts from the deviated communities.

For each POD community, we further computed the weighted clustering coefficient of the keyword co-occurrence networks constructed from offensive and non-offensive posts separately, following the definition in Section 3.2.3. As shown in Table 4, the offensive network consistently yields a higher weighted clustering coefficient than its non-offensive counterpart for every community. This indicates that, in our case, offensive discussions are more structurally cohesive than non-offensive ones.

**Figure 6:**
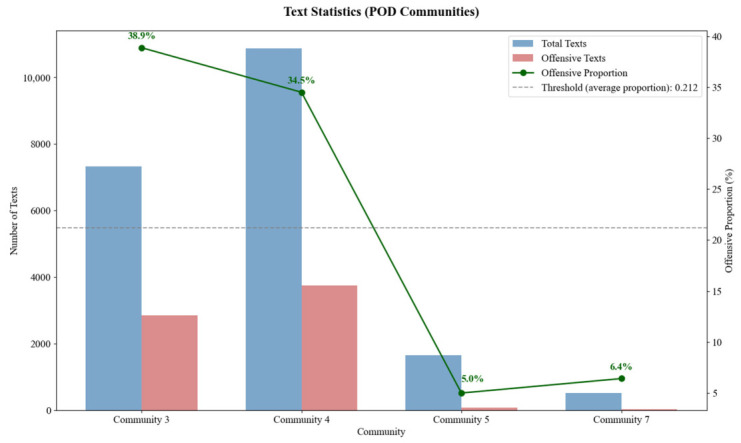
Statistics of text characteristics in POD communities.

According to the Pareto principle, prioritizing the control of the more severely problematic groups can effectively manage overall public opinion. As illustrated in Figure 6, Communities 3 and 4 contain a much higher proportion of offensive texts than Communities 5 and 7. Given the substantial gap in offensive text proportions between Communities 3/4 and Communities 5/7, no further expert judgment is invoked. Using Equation (6), the threshold is calculated to be 0.212. Communities 3 and 4 exceed the threshold and are identified as offensive-boundary communities ($Q_{POD}^{1}$), while Communities 5 and 7 fall below the threshold and are classified as non-offensive-boundary communities ($Q_{POD}^{0}$).

### 4.3. Simulation and Model Validation

#### 4.3.1. Parameter Settings

A simulation experiment was conducted to reproduce the POD scenario involving group negative emotions during the “10·28” Chongqing bus plunge incident. Due to the large scale of the user group in the real data, it is extremely challenging to quantitatively handle user relationships and individual characteristics. Therefore, the simulation appropriately simplifies the real scenario as follows: (1) The number of people remains constant; (2) individuals are heterogeneous; and (3) the openness of users to external opinions and their sensitivity to public opinion deviation are both considered external stimuli. This paper assumes that users’ openness to external views is equivalent to their sensitivity to public opinion deviation. The openness of users to external opinions and their sensitivity to POD are both treated as external stimuli. In this study, we assume that a user’s openness to external views is equivalent to their sensitivity to POD.

Suppose that during this public opinion incident, there are n individuals in the communities exhibiting opinion deviation. Consistent with assumption (1) and for simulation efficiency, we set n = 200. In the actual case, the proportions of texts from POD communities with and without offensive boundaries are 89% and 11%, respectively. Accordingly, in the simulation, 89% of the individuals belong to POD communities with offensive boundaries, and the remaining 11% belong to those without.

At each time step, an individuals’ negative emotion is influenced by multiple factors: the severity of POD, individuals’ sensitivity to such deviation, their initial negative emotions, and the negative emotions of neighbors. Each simulation time step corresponds to a 12 h real-world interval. Time step *t* = 1 corresponds to the period from 0:00 to 12:00 on 28 October 2018. From 28 October 2018 to 7 November 2018, there are 22 such intervals; thus, $t_{max}$ was set to 22.

Following assumption (2), the diagonal entries $b_{ii}$ of the POD sensitivity matrix *B* are randomly drawn from the interval [0, 1]. The values of the POD severity matrix $S_{t}$ are obtained from the multi-stage quantification model (Equations (7)–(10)), as shown in Table 5. According to assumption (3), an individual’s openness to neighbors’ negative emotions is set equal to their sensitivity to POD, i.e., $a_{ii}=b_{ii}$. In the influence weight matrix *W*, we set $w_{ii}=1\;-\;a_{ii}$. For j ≠ i, each $w_{ij}$ is initially assigned a random value $w_{ij}^{*}\;$∈ [0, 1] and then normalized so that each row of *W* sums to 1: $w_{ij}=w_{ij}^{*}\times \frac{1\;-\;w_{ii}}{\sum_{j\neq i}w_{ij}^{*}}$. The key simulation parameters for updating negative emotion values at each time step are summarized in Table 6.

#### 4.3.2. Simulation Results

**Figure 7:**
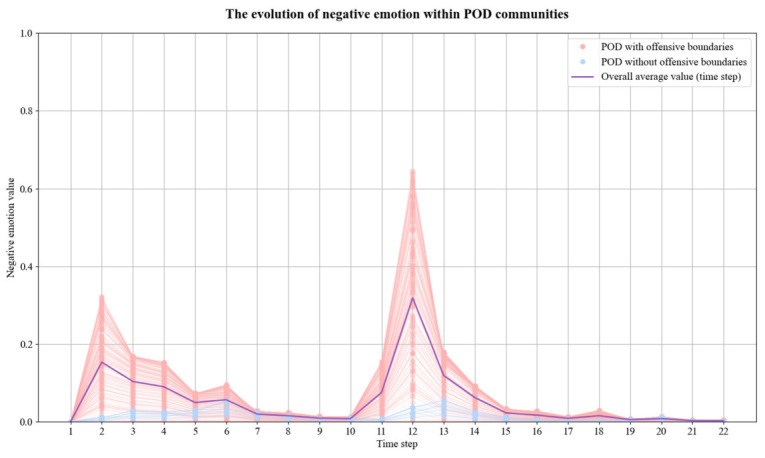
The evolution of negative emotions within POD communities.

Given that the acquired data on POD severity already incorporate the intervention effects present in real-world scenarios, we conducted a simulation experiment using Equation (11) to replicate the observed trend of negative emotion evolution within actual POD communities. The results are shown in Figure 7. The red line and the blue line represent the evolution of negative emotions among individuals in offensive and non-offensive POD communities, respectively. Under the same level of POD severity, individuals exhibit different trajectories: some show a sharp increase in negative emotion, while others experience only a mild rise. The purple line indicates the evolution of the average negative emotion value across all individuals.

#### 4.3.3. Model Validation

**Figure 8:**
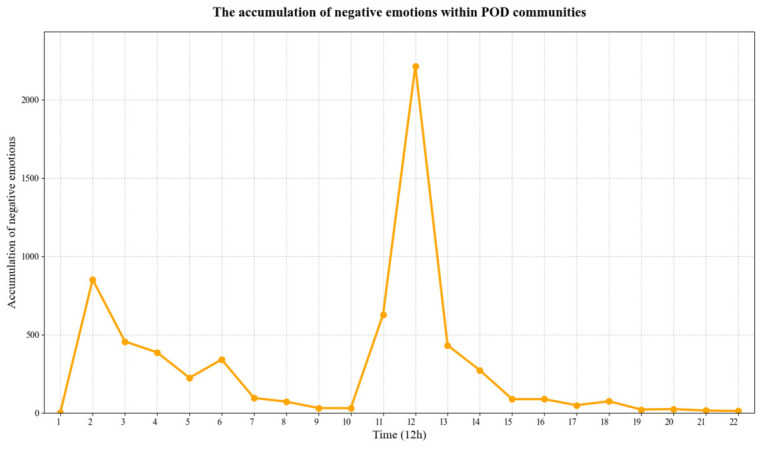
The accumulation of negative emotions within POD communities.

Figure 8 presents real data on the accumulation of negative emotions in POD communities during the Chongqing bus plunge incident. It is important to note that the negative emotion accumulation at each stage, as derived from the fuzzy inference rules defined in this paper, is inherently independent of the number of posts and comments in that stage used in the POD severity quantification model. Specifically, a text receives a positive negative-emotion value only when its SnowNLP sentiment score falls within a predefined interval.

In this study, the Pearson correlation coefficient is employed to calculate the correlation. This coefficient is obtained by dividing the covariance of the average negative emotion value at each time step in the simulation experiment and the negative emotion accumulation within POD communities in the real world by the product of their standard deviations (Xie et al., 2023). This calculation serves to evaluate the similarity of the negative emotion change trends between the simulated data and the real data. The results are presented in Table 7.

This finding reveals a strong positive correlation between the simulated and real data, indicating that the POD F-J model accurately predicts the evolutionary trend of negative emotions within actual POD communities. Therefore, it can be used to simulate intervention strategies in real-world scenarios.

#### 4.3.4. Ablation Experiments

To verify the validity of the parameters of the proposed model, we conduct ablation experiments on the POD severity quantification model and the POD F-J model.

##### Effects of Severity Quantification Components

**Figure 9:**
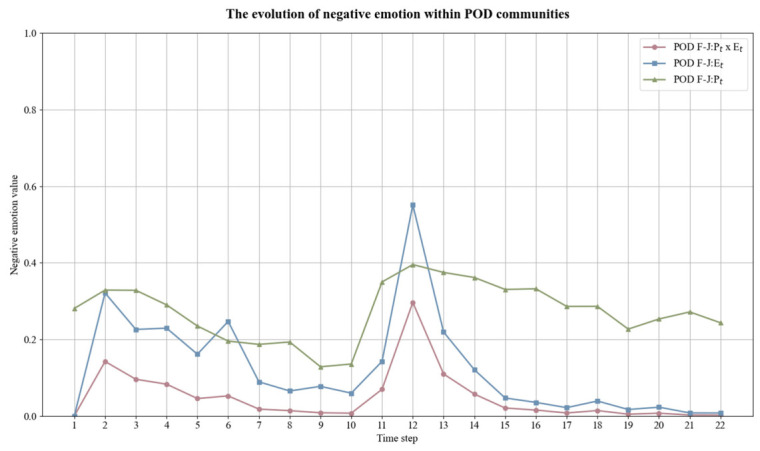
Ablation experiments on the POD severity quantification model.

First, an ablation experiment was conducted on the POD severity quantification model, as shown in Figure 9, with the results presented in Table 8. The findings reveal that the proposed model, which considers both *P_t_* ($P_{1,t}\;\mathrm{and}\;P_{0,t}$) and *E_t_*, achieves the best fit in the simulation experiment, outperforming single-dimension models. The model using only *E_t_* ranks second and performs comparably to the optimal model, whereas the model using only *P_t_* performs relatively poorly. This indicates that the number of posts and comments from POD communities is more informative for quantifying POD severity than their proportion. Nevertheless, including the proportion still improves the model’s ability to capture POD severity. Although public opinion heat measures used in previous studies can describe the severity of opinion deviation to some extent, the POD severity quantification model proposed in this paper is superior.

##### Effects of the POD F-J Model Components

**Figure 10:**
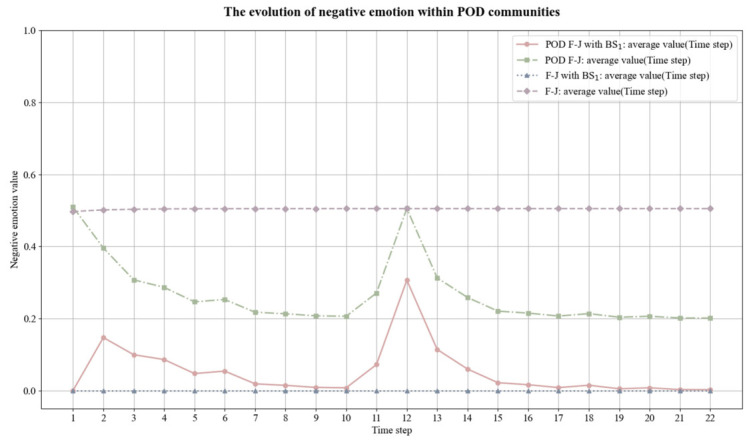
Ablation experiments on the POD F-J model.

Next, we conducted ablation experiments on the POD F-J model and the initial value setting method, as shown in Figure 10, with the results presented in Table 9. The results indicate that the classical F-J model poorly captures the phenomenon of public opinion deviation and cannot adequately fit the evolution of negative emotions in deviation communities during public opinion development. However, after augmenting the F-J model with the term *BS_t_*—which represents the negative emotional stimulation from perceiving public opinion deviation—the model’s fit to the negative emotion evolution process improves significantly. Furthermore, setting the initial negative emotion value to *BS*_1_ further enhances the fitting accuracy. It should be noted that “NaN” appears because the Pearson correlation coefficient cannot be calculated for the F-J model with *BS*_1_. This occurs because *BS*_1_ yields an all-zero matrix, and the F-J model does not account for subsequent negative emotional stimuli from public opinion deviation. Consequently, the average negative emotion remains zero, making it impossible to compute the correlation coefficient.

In summary, *BS*_1_ helps determine the initial negative emotion values of the deviation group, while the combination of *BS_t_* matrices enables the model to capture the negative emotional stimulation caused by public opinion deviation on users. In a public opinion deviation scenario, the classical F-J model can only simulate a group that does not perceive the negative emotional pressure of deviation—for example, individuals who are completely detached from the event or so insensitive to the negative emotional stimulus that its effect can be ignored. Therefore, the classical F-J model is unsuitable for predicting the evolution of negative emotions within deviation communities. In contrast, the POD F-J model with *BS*_1_ achieves the best predictive performance.

### 4.4. Intervention Effect Simulation and Timing Analysis

#### 4.4.1. Comparative Analysis of Intervention Strategies

In highly intricate emergency situations, it is usually challenging to obtain and implement intervention strategies that excel in both immediate intervention strength and intervention retention. In this study, intervention simulation experiments are conducted using “guiding,” “compulsory,” and “mixed” strategies. Based on the theoretical foundations of an existing study—a systematic investigation of China’s government practices in online public opinion governance (Ren & Chao, 2021), we assign appropriate parameter sets to different intervention strategies according to their relative characteristics, as shown in Table 10.

In the context of POD scenarios during emergencies, the number of effective interventions is usually limited. We define the maximum number of such interventions as *N* ∈ {0, 1, 2, …, 10}. For the intervention strategy simulation experiments, we use the IPOD F-J model. Compared with Table 5, the parameters now include the intervention effect *I_t_*, the immediate intervention strength *Strength*, the intervention retention *Retention*, the efficiency-oriented intervention timing set $\left\{\tau \right\}$, the upper limit on the number of effective interventions *N*, and the emotional rebound index α (set to 2.0 in the experiments; see Section 4.4.3 for a sensitivity analysis of α).

**Figure 11:**
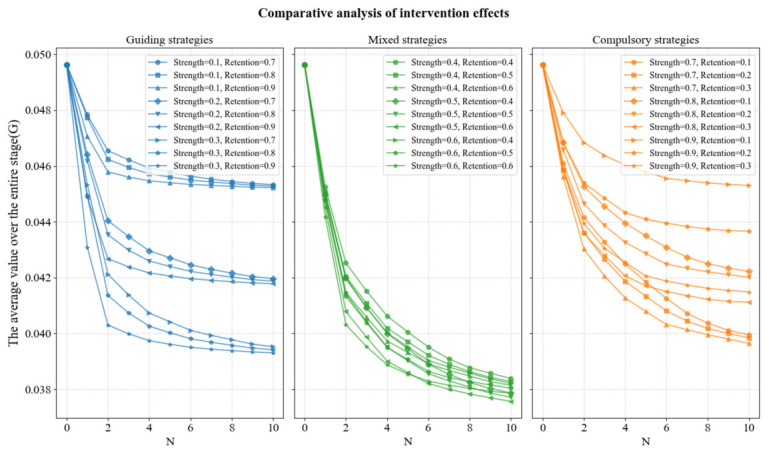
Comparative analysis of intervention effects.

Using Equations (12)–(14), we simulate group negative emotion interventions under different strategies and analyze how varying the intervention parameter combinations affects the intervention effect, as shown in Figure 11. The results indicate that, when the greedy algorithm is used to select intervention timings, the average negative emotion value over the whole period generally decreases as the maximum number of effective interventions *N* increases, meaning that the intervention effect improves.

**Figure 12:**
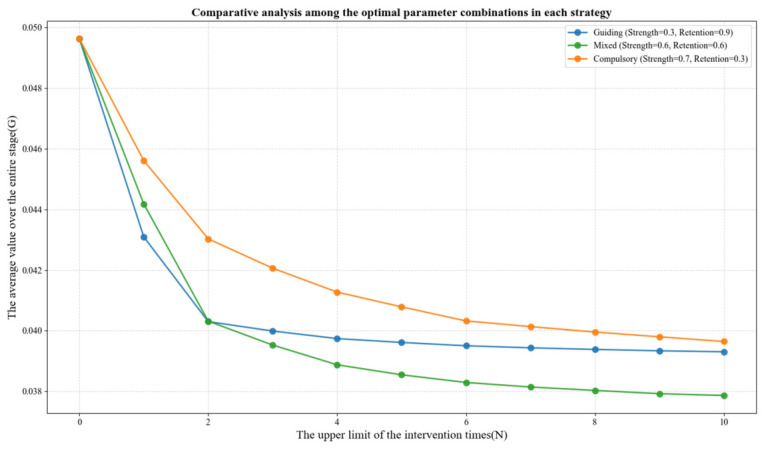
Comparative analysis of the intervention effects among the optimal parameter combinations in each strategy.

Moreover, we performed a comparative analysis of the intervention effects among the optimal parameter combinations for each strategy. As shown in Figure 12, we compared the intervention effects of the optimal strategies across the three types. Under the premise that the efficiency-oriented intervention timing is determined, we found that when the number of effective interventions is extremely limited (i.e., *N* = 1), the guiding intervention strategy yields the best outcome. However, as the number of effective interventions increases, the mixed intervention strategy gradually outperforms the guiding strategy, indicating that when sufficient interventions are allowed, the intervention strength can be appropriately increased. It should be noted, however, that the compulsory intervention strategy never achieves the best performance.

This conclusion aligns closely with the qualitative findings of existing research (Ren & Chao, 2021), which indicate that guiding and mixed intervention strategies are gaining importance in China’s online public opinion governance. By converting qualitative descriptions into systematic parameter settings, our quantitative mathematical model reproduces the actual regularities of online public opinion governance. Furthermore, it offers a quantitative tool for opinion management in deviation communities, allowing for the analysis of suitable scenarios for different intervention strategies.

When implementing compulsory strategies in real-world scenarios, public responses with different types of fine-grained negative emotions may vary. According to the theory of psychological reactance, when dealing with negative emotions characterized by high dominance—such as anger (James & Albert, 1974)—compulsory intervention strategies may be perceived as an infringement on individual dignity; this perception may trigger a group-level “backfire effect.” That is to say, when a false piece of information is corrected and this correction contradicts people’s original views, it may instead deepen people’s trust in that false information and increase the difficulty of public opinion control. On the other hand, according to learned helplessness theory for negative emotions characterized by compliance—such as anxiety (James & Albert, 1974)—the loss of perceived control experienced by their holders under compulsory intervention strategies will strengthen attributions regarding “uncontrollable results,” triggering self-silence and possible psychological crises. Furthermore, according to the principle of proportionality in public law—which is used to restrict the intervention of public power in citizens’ basic rights—for instance, compulsory strategies such as deleting posts, silencing users, and suspending accounts directly affect citizens’ right to freedom of speech and may excessively infringe upon individual rights, thereby triggering ethical crises. Therefore, compulsory strategies have limitations in practical application.

In addition, it is important to note that the principle of reasonable accommodation should also be considered in actual platform governance. Reasonable accommodation means “necessary and appropriate modification and adjustments not imposing a disproportionate or undue burden, where needed in a particular case” (United Nations Human Rights Office of the High Commissioner, 2006). This principle emphasizes providing personalized, immediate accommodations for specific disadvantaged groups to remove structural barriers and achieve substantive equality.

In the present case, POD communities with offensive boundaries generated a large number of offensive statements that shifted responsibility onto female drivers and attacked women using negative stereotypes. In response, the principle of reasonable accommodation offers a complementary approach to platform governance beyond content removal or user silencing—namely, information protection. Specifically, platforms can fulfill their reasonable accommodation obligations by adopting the following measures: (1) prioritize fact-checking algorithms to limit the visibility of or annotate speech that explicitly contains gender-based insults or scapegoating attributions; simultaneously, reduce the exposure of texts that disclose female drivers’ personal information (e.g., real names, photos); (2) provide counter-narratives that oppose offensive framing, and appropriately increase the delivery of anti-gender-discrimination educational content in relevant topic feeds, so as to balance the information ecosystem and alleviate gender bias in public discourse.

Platform governance should adopt differentiated strategies depending on whether a POD community has offensive boundaries: for non-offensive communities, the focus should be on managing informational noise; for offensive communities, information protection and anti-discrimination education should be taken into account to achieve the best governance outcomes.

#### 4.4.2. Simulation of Intervention Timings

**Figure 13:**
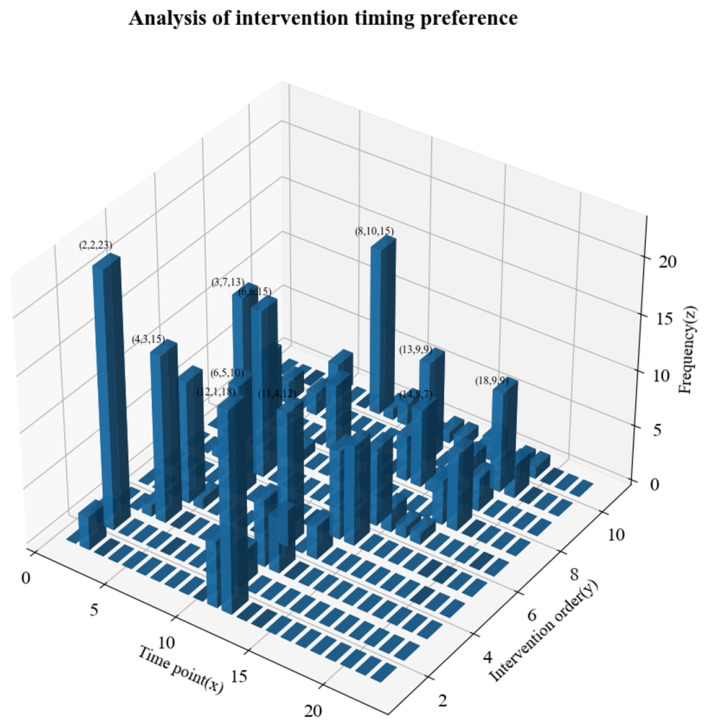
Analysis of intervention timing preference.

Using the greedy algorithm, this study accurately determines the efficiency-oriented intervention timings. Figure 13 presents an analysis of intervention timing preferences across 27 intervention strategies belonging to three types. Although the priority of intervention timing varies among strategies, most preferentially select time steps 12 and 2. Notably, time step 12 marks the peak of POD severity in offensive communities, highlighting the need to promptly identify such extreme points and intervene to mitigate group negative emotions. Time step 2 corresponds to the onset of POD, suggesting that early intervention plays a key role in managing public sentiment during opinion deviation.

#### 4.4.3. Sensitivity Analysis of the Rebound Index

**Figure 14:**
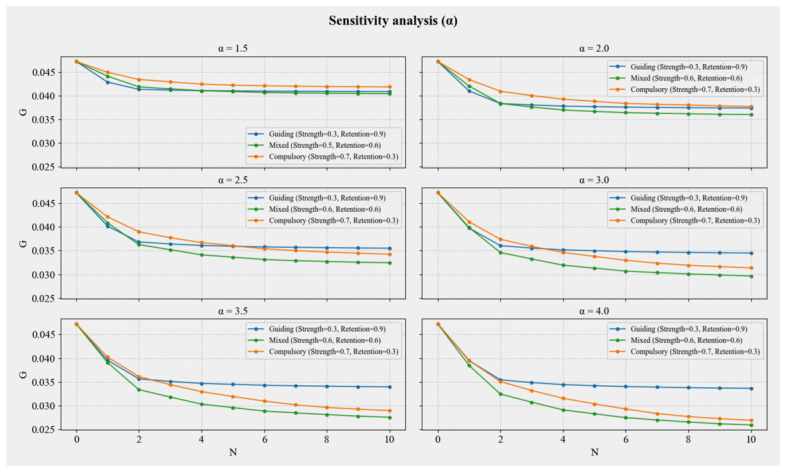
Sensitivity analysis.

A sensitivity analysis was conducted for different values of α, specifically 1.5, 2.0, 2.5, 3.0, 3.5, and 4.0. The results are shown in Figure 14. When public sensitivity to intervention is high (α = 1.5, 2.0, 2.5), the guiding intervention strategy performs best when the number of effective interventions is extremely limited (*N* = 1). As the number of interventions increases, the mixed strategy gradually becomes the better option. When public sensitivity to intervention is low (α = 3.0, 3.5, 4.0), the mixed strategy is the optimal choice. This suggests that intervention strength can be moderately increased in such cases, but not at the expense of intervention persistence. In summary, intervention strategies should be selected based on a combination of factors, including public sensitivity to intervention and the number of effective interventions available.

## 5. Conclusions

This research analyzes the impact of public opinion deviation in the post-truth era on group negative emotions. It constructs an identification model for POD communities, a multi-stage model for quantifying POD severity, a group negative emotion evolution model (the POD F-J model), and an intervention-oriented model (the IPOD F-J model). At the information level, it reveals the intrinsic correlation between POD severity and group negative emotions, confirming the necessity of targeted intervention. Using simulation experiments, this study compares, for the first time, three intervention strategies classified by degree of compulsion in a POD scenario, examining their optimal effects and preferred intervention timings. The results show that different intervention strategies should be adopted depending on the situation (e.g., the public’s degree of resistance to intervention and the number of effective interventions available). Furthermore, the onset of public opinion deviation and the moment of peak deviation severity are identified as priority intervention points. These findings highlight that selecting appropriate intervention strategies and timings is essential for managing group negative emotions with a limited number of effective interventions.

The method proposed in this paper is generalizable. The identification of core issues depends on the specific context. For example, in the case study presented here, ‘attributing responsibility’ did not emerge as a core issue, but that does not mean it cannot become one in other contexts. The fine-tuning dataset used for the BERT model in this paper can also be replaced with corresponding data based on specific events, such as community division or political conflict.

The main contribution of this paper lies in using readily available social media data to provide decision support for governing group negative emotions in POD scenarios. Specifically, it achieves the identification of POD communities in emergencies, the multi-stage quantification of POD severity, the simulation of negative emotion evolution and intervention effects within such communities, and the analysis of intervention timing preferences—that is, quantifying when to intervene and how to combine different strategies under a limited number of effective interventions. The real-world case study validates the applicability of the proposed model. Nevertheless, the model still has considerable room for improvement.

It should be noted that this study addresses opinion deviation from the perspective of emergency decision support and may not be directly generalizable to other contexts. In future work, we can conduct multi-case analyses of various public opinion deviation (POD) events. We will also investigate the evolution of fine-grained negative emotions in opinion deviation scenarios and the simulation of interventions. Furthermore, the principle of “reasonable accommodation,” discussed in the intervention experiment section of this paper, presents a valuable direction for future research: mathematically embedding this principle could help explore the interactive dynamics between offensive opinion deviation communities and the victims.

## Figures and Tables

**Table 1:** Fuzzy rule base.

Fuzzy Mapping Rules	Sentiment Score	Negative Emotion Value
${\mathrm{Rule}}_{1}$ Extremely negative	{0.00, 0.00, 0.05}	{0.85, 0.95, 1.00}
${\mathrm{Rule}}_{2}$ Very negative	{0.00, 0.05, 0.10}	{0.70, 0.85, 0.95}
${\mathrm{Rule}}_{3}$ Moderately negative	{0.05, 0.10, 0.15}	{0.55, 0.70, 0.85}
${\mathrm{Rule}}_{4}$ Negative	{0.10, 0.15, 0.20}	{0.40, 0.55, 0.70}
${\mathrm{Rule}}_{5}$ Slightly negative	{0.15, 0.20, 0.25}	{0.25, 0.40, 0.55}
${\mathrm{Rule}}_{6}$ Very slightly negative	{0.20, 0.25, 0.30}	{0.10, 0.25, 0.40}
${\mathrm{Rule}}_{7}$ Barely negative	{0.25, 0.30, 0.35}	{0.05, 0.10, 0.25}
${\mathrm{Rule}}_{8}$ Almost Neural	{0.30, 0.35, 0.40}	{0.00, 0.05, 0.10}

**Table 2:** Community keywords.

Community	Keywords
1	重庆 (Chongqing), 公交 (bus), 关注 (attention), 悲剧 (tragedy), 孩子 (child), 文章 (article), 逃生 (escape)
2	公交车 (bus), 视频 (video), 万州 (Wanzhou), 现场 (scene), 行驶 (driving car), 小轿车 (car), 调查 (investigation), 长江 (the Yangtze River), 护栏 (guardrail), 行车 (driving), 监控 (surveillance), 记录 仪 (recorder), 撞击 (crash), 坠入 (fall into), 客车 (passenger vehicle), 江中 (river), 位置 (location)
3	事故 (accident), 女司机 (female driver), 新闻 (news), 逆行 (driving in the wrong direction), 媒体 (media), 大巴车 (bus), 网友 (netizens), 开车 (driving car), 小车 (car), 轿车 (sedan), 评论 (comment), 控制 (control), 造谣 (spreading rumors), 大巴 (bus), 道歉 (apology), 键盘 (keyboard), 微笑 (smile), 网络 (internet), 官方 (official), 警方 (police), 通报 (notice), 高跟鞋 (high heels), 报道 (report), 节奏 (rhythm), 谣言 (rumor)
4	司机 (driver), 乘客 (passenger), 原因 (reason), 情况 (situation), 车辆 (vehicle), 责任 (responsibility), 方向盘 (steering wheel), 社会 (society), 驾驶员 (driver), 驾驶 (driving), 公布 (announce), 失控 (out of control), 互殴 (fight with each other), 方向 (direction), 泼妇 (shrew), 素质 (quality), 下车 (get off the vehicle), 错过 (miss), 法律 (law), 冲动 (impulse), 女人 (woman), 情绪 (emotion), 冷漠 (indifference), 争执 (dispute), 刹车 (brake), 停车 (stop the vehicle), 制止 (stop), 突发 (sudden), 抢夺 (snatch), 涉嫌犯罪 (suspected of crime)
5	家属 (family members), 打捞 (salvage), 遇难者 (victims), 发现 (discovery), 遗体 (remains), 找到 (found), 家人 (family), 潜水员 (diver), 赔偿 (compensation), 律师 (lawyer)
6	希望 (hope), 蜡烛 (candle), 逝者 (the deceased), 生命 (life), 无辜 (innocent), 时间 (time), 安息 (rest in peace), 救援 (rescue), 人员 (personnel), 可怜 (pitiful), 伤心 (sad), 祈祷 (pray), 奇迹 (miracle), 救援队 (rescue team), 游泳 (swimming)
7	中国 (China), 地点 (location), 长沙 (Changsha), 音乐 (music), 郴州 (Chenzhou)

**Table 3:** The identification results of POD communities.

q	Community	Degree of Topic Deviation	The Value Used for Comparison Is the Mean.	Result
1	“重庆 (Chongqing)-公交 (bus)-关注 (attention)-悲剧 (tragedy)” Community 1	0.4948	0.8724	Normal community
2	“公交车 (bus)-视频 (video)-万州 (Wanzhou)-现场 (scene)” Community 2	0.8562	0.8724	Normal community
3	“事故 (accident)-女司机 (female driver)-新闻 (news)-逆行 (driving in the wrong direction)” Community 3	1.0239	0.8724	POD community
4	“司机 (driver)-乘客 (passenger)-原因 (reason)-情况 (situation)” Community 4	0.9916	0.8724	POD community
5	“家属 (family members)-打捞 (salvage)-遇难者 (victims)-发现 (discovery)” Community 5	0.9540	0.8724	POD community
6	“希望 (hope)-蜡烛 (candle)-逝者 (the deceased)-生命 (life)” Community 6	0.7078	0.8724	Normal community
7	“中国 (China)-地点 (location)-长沙 (Changsha)-音乐 (music)” Community 7	1.0783	0.8724	POD community

**Table 4:** Weighted Clustering Coefficient.

Community	${\bar{C}}_{off,q}^{w}$	${\bar{C}}_{non,q}^{w}$	Comparison
3	0.4165	0.4010	>
4	0.4113	0.4035	>
5	0.4528	0.4284	>
7	0.4775	0.4645	>

**Table 5:** The severity of public opinion deviation at each stage.

Time (12 h)	1	2	3	4	5	6	7	8	9	10	11
*S_t_* $\left(Q_{POD}^{1}\right)$	0.000	0.322	0.168	0.153	0.071	0.095	0.020	0.023	0.013	0.012	0.155
*S_t_* $\left(Q_{POD}^{0}\right)$	0.000	0.013	0.009	0.012	0.022	0.056	0.017	0.014	0.006	0.010	0.006
**Time (12 h)**	**12**	**13**	**14**	**15**	**16**	**17**	**18**	**19**	**20**	**21**	**22**
*S_t_* $\left(Q_{POD}^{1}\right)$	0.646	0.146	0.089	0.026	0.027	0.012	0.029	0.005	0.013	0.003	0.004
*S_t_* $\left(Q_{POD}^{0}\right)$	0.035	0.010	0.005	0.002	0.002	0.001	0.002	0.004	0.013	0.000	0.000

**Table 6:** The important simulation parameters of evolutionary process.

The Parameters Directly Involved in the Simulation Experiment	Value Description
*X_t_*	The negative emotion value at each time step (except the initial time step) is obtained using the improved F-J model (POD F-J model) proposed in this paper.
*X*_1_	The initial negative emotion value is derived from the POD severity at the initial time step and the individual’s sensitivity to POD.
*S_t_*	The POD severity in the *t*-th time period is obtained by processing real data with the POD severity quantification model proposed in this paper.
*B*	In accordance with assumption (2), the POD sensitivity value *b_ii_* of individual *i* is randomly selected from the interval [0, 1].
*A*	The openness of individual *i*, *a_ii_*, is set equal to *b_ii_*; *a_ii_* = *b_ii_* in accordance with assumption (3).
*W*	The influence weight matrix is determined by the openness matrix: $w_{ii}=1\;-\;a_{ii}$. For j ≠ i, each $w_{ij}$ is initially a random value in [0, 1] and then normalized so that each row sums to 1.
*n*	In accordance with assumption (1) and for simulation efficiency, the number of individuals is set to 200.
Parameters indirectly involved in the simulation experiment	Value Description
$P_{1,t}\;\mathrm{and}\;P_{0,t}$	These values are obtained from real data processed by the POD community identification model.
*E_t_*	The value of *E_t_* is obtained through the real data.

**Table 7:** Pearson Correlation Coefficient.

Model	Pearson Correlation Coefficient	*p*-Value
POD F-J model	0.9758	1.1174 × 10^−14^

**Table 8:** Pearson correlation coefficients for the ablation experiments on the POD severity quantification model.

Model	POD Severity Quantification Model	Pearson Correlation Coefficient	*p*-Value
POD F-J	*P_t_* × *E_t_*	0.9762	9.4201 × 10^−15^
POD F-J	*E_t_*	0.9245	7.8933 × 10^−10^
POD F-J	*P_t_*	0.5456	0.0086

**Table 9:** Pearson correlation coefficients for the ablation experiments on the POD F-J model.

Model	X_1_	Pearson Correlation Coefficient	*p*-Value
POD F-J	*BS*_1_	0.9758	1.1240 × 10^−14^
POD F-J	Random	0.6884	0.0004
F-J	*BS*_1_	NaN	NaN
F-J	Random	0.0045	0.9840

**Table 10:** Intervention strategy parameter settings.

Types of Intervention Strategies	Degree of Compulsion	Set of *Strength* Values	Set of *Retention* Values
Guiding	low	{0.1, 0.2, 0.3}	{0.7, 0.8, 0.9}
Compulsory	high	{0.7, 0.8, 0.9}	{0.1, 0.2, 0.3}
Mixed	medium	{0.4, 0.5, 0.6}	{0.4, 0.5, 0.6}

## Data Availability

The data that support the findings of this study are available from the corresponding author upon reasonable request.

## Appendix A

This section compares the greedy algorithm with the exhaustive enumeration method. The absolute difference is used to measure the gap in the G value, and the Jaccard similarity coefficient is used to quantify the overlap between the intervention time sets selected by the two algorithms, as specified in Equations (A1) and (A2).

(A1) $$absolute\;error=|G(\{\tau {\}}_{\mathrm{greedy}})-G(\{\tau {\}}_{\mathrm{optimal}})|$$

(A2) $$J\;\left(\{\tau {\}}_{\mathrm{greedy}},\{\tau {\}}_{\mathrm{optimal}}\right)=\frac{\left|\{\tau {\}}_{\mathrm{greedy}}\cap \{\tau {\}}_{\mathrm{optimal}}\right|}{\left|\{\tau {\}}_{\mathrm{greedy}}\cup \{\tau {\}}_{\mathrm{optimal}}\right|}$$

Given the constraints on computational resources, we conducted experiments for *N* = 0, 1, 2, 3, 4, 5 as an illustrative case. The results show that across all types of intervention strategies, the average absolute difference between the greedy algorithm and the exhaustive enumeration method is 0.0000061176, and the average Jaccard similarity coefficient is 0.9730452675. This indicates that although the intervention time sets and the corresponding intervention effects obtained by the greedy algorithm and the exhaustive enumeration method are not identical, the differences are negligibly small and therefore acceptable.

Considering that time urgency and limited computational resources are the core characteristics of decision-making in emergency scenarios, the greedy algorithm—with its transparency, low computational cost, and ease of interpretation—has become a commonly used heuristic for selecting intervention timings in such contexts (Q. Zhang et al., 2025). In contrast, methods that can guarantee a globally optimal solution, such as exhaustive enumeration, are difficult to apply in emergency settings due to their high computational cost and long execution time.

Moreover, in emergency scenarios, the maximum allowable number of interventions (N) is highly likely to change. The greedy algorithm can ensure a relatively stable set of intervention timings despite such changes. Exhaustive enumeration, on the other hand, may completely overturn previous decisions in order to maximize intervention effectiveness, which could undermine the coordination efficiency of emergency response. At the same time, frequent reversals of decisions—akin to “changing orders overnight”—may also damage government credibility.
